# EADC Values in Diagnosis of Renal Lesions by 3.0 T Diffusion-Weighted Magnetic Resonance Imaging: Compared with the ADC Values

**DOI:** 10.1007/s00723-012-0376-z

**Published:** 2012-07-05

**Authors:** Yue-Lang Zhang, Bo-Lang Yu, Juan Ren, Kai Qu, Ke Wang, Yong-Qian Qiang, Chen-Xia Li, Xing-Wang Sun

**Affiliations:** 1 Department of Imaging, First Affiliated Hospital, Medicine School of Xi’an Jiaotong University, Xi’an, 710061 Shaanxi People’s Republic of China; 2 Department of Radiotherapy Oncology, First Affiliated Hospital, Medicine School of Xi’an Jiaotong University, 277 Yanta West Road, Xi’an, 710061 Shaanxi People’s Republic of China

## Abstract

Exponential apparent diffusion coefficient (EADC) is an indicator of diffusion-weighted imaging (DWI) and reflects the pathological changes of tissues quantitatively. However, no study has been investigated in the space-occupying kidney disease using EADC values. This study aims to evaluate the diagnostic role of EADC values at a high magnetic field strength (3.0 T) in kidney neoplastic lesions, compared with that of the ADC values. Ninety patients with suspected renal tumors (including 101 suspected renal lesions) and 20 healthy volunteers were performed MRI scanning. Diffusion-weighted imaging was performed with a single-shot spin-echo echo-planar imaging (SE-EPI) sequence at a diffusion gradient of *b* = 500 s/mm^2^. We found renal cell carcinoma (RCC) can be distinguished from angiomyolipoma, and clear cell carcinoma can be distinguished from non-clear cell carcinoma by EADC value. There was significant difference in overall EADC values between renal cell carcinoma (0.150 ± 0.059) and angiomyolipoma (0.270 ± 0.108) when *b* value was 500 s/mm^2^. When receiver operating characteristic (ROC) was higher than 0.192, the sensitivity and specificity of EADC value of renal cell carcinoma were 84.6 and 81.1 %, respectively. In conclusion, EADC map shows the internal structure of the kidney tumor more intuitively than the ADC map dose, and is also in line with the observation habits of the clinicians. EADC can be used as an effective imaging method for tumor diagnosis.

## Introduction

Computed tomography (CT) and magnetic resonance imaging (MRI) are routinely applied in diagnosis of kidney disease to distinguish benign tumors from malignant tumors, and to determine the stage and grade of tumors [1–3]. However, it is difficult to distinguish benign and malignant tumors when the images are atypical or the lesions in early stage are small, which causes 10.2–21.5 % benign tumors were removed as malignant tumors [4–6]. CT contrast agents can cause adverse reaction or renal toxicity [7, 8], while MRI contrast agents can increase the risk of nephrogenic systemic fibrosis with renal insufficiency [9–11]. Therefore, researches on non-enhanced functional MRI will provide important information for safer diagnostic imaging in kidney cancer.

Diffusion-weighted imaging (DWI) is able to evaluate random movement of water molecules through the diffusion of water molecules in vivo, thus providing information on the spatial structure of the tissues. Apparent diffusion coefficient (ADC) can quantitatively reflect the pathological changes of tissues, and is very useful in clinical diagnosis of central nervous system diseases, especially in hyperacute cerebral infarction [12–14]. DWI can also be used as a clinical application in kidney lesions and used to evaluate the ADC value in the differentiating diagnosis among various subgroups of renal masses [15], and has a certain value in classification and grading of renal cell carcinoma [16, 17].

Diffusion-weighted imaging shows high signal in diffusion-restricted tissues, however, tissues with long repetition time (TR) show high signal even in the absence of diffusion barriers, which is mainly caused by the T2 effect. Though ADC maps eliminate the T2 effect, the diffusion-restricted tissue shows low signal intensity, while diffusion-free tissue shows high signal intensity. Exponential apparent diffusion coefficient (EADC) is a new DWI quantitative indicator. Compared with ADC value, the EADC value not only eliminates the T2 transmission effect, but also retains the characteristics of the DWI map signals. Therefore, the substantial part of tumor shows high signal intensity, which highlights the lesion more efficiently and makes it easier to identify the structure within the lesion. EADC map is in line with the habits that the clinicians observe lesions, and is also able to obtain quantitative data.

Diffusion-limited tissues showed high signal intensity on DWI, and showed low signal on the ADC map, with low ADC value. While free diffusion tissues showed low signal intensity on DWI, with high ADC signal intensity and high ADC value. That means diffusion-limited tissues show high signal intensities with high EADC values, while free diffusion tissues show low signal intensities with low EADC values. EADC value displays the movement of water molecules within the tissue, and its applications in central nervous system disease, breast disease, and whole-body diffusion studies have been reported [18–20], but no study has been found in the space-occupying kidney disease. This study aims to compare the diagnostic effect of EADC and ADC values at high magnetic field strength 3.0 T in kidney neoplastic lesions.

## Materials and Methods

### The Study Subject

The protocol was discussed and approved by the Ethics Committee in the first hospital of Xi’an Jiaotong University. All patients were informed about their participation in this study and signed written consent was acquired. Healthy volunteers and patients with suspected renal tumors were recruited in this study between December 2010 and November 2011. The inclusion criteria for controls were: (1) no clinical symptoms of urinary system diseases; (2) normal urine and renal function; (3) no previous history of kidney disease; (4) no history of hypertension, diabetes or other diseases; (5) no nephrotoxic drugs taken recently; (6) normal MRI images. The inclusion criteria for patients were: (1) kidney neoplastic lesions, including renal cell carcinoma (RCC), renal angiomyolipoma and simple renal cysts, were identified by B-ultrasonography, CT or MRI scanning; (2) patients with renal tumors who were planed to underwent surgical resection; (3) angiomyolipoma and simple renal cysts with typical imaging findings and no change was found in follow-up. The follow-up MRI was performed once every 6 months; (4) cooperation of patients with the inspection. Exclusion criteria: (1) patients with absolute contraindications towards MRI scanning; (2) patients who did not been confirmed by surgery or puncture and did not underwent follow-up; (3) patients whose lesion size and signal intensity changed during follow-up.

### Scanning Method

All scans were performed using 3.0 T MR (Signa HDx 3.0T, GE Medical Systems) with 8US TORSOPA coil. All subjects had fasted for 6 h and were trained to hold breath at the end of expiratory before scanning to minimize inter-subject and intra-subject variation. The routine renal mass MR imaging were carried out according to the manufacturer’s protocol. Briefly, transverse and coronal T2-weighted single-shot fast spin-echo sequences, and T1-weighted 3D breath-hold in-phase and out-of-phase sequence were performed.

Three-dimensional enhanced MRI scanning with fat-saturated T1-weighted dynamic contrast material was performed during suspended respiration. Magevist (Gadopentetic Acid Dimeglumine Salt Injection, Bayer Schering Pharma AG, Berlin, Germany) (15 mL) was injected intravenously at a rate of 2 mL/sec using a power injector (Spectris; MEDRAD, Warrendale, USA), followed by a 20-mL saline flush. LAVA-Mask scan (precontrast) was performed before enhanced scanning. Dynamic contrast-enhanced MRI was performed in the transverse plane during the cortical phase, medullary phase and delayed phase, and the scan time in each breath-hold was 14–16 s.

Transverse breath-hold diffusion-weighted images were obtained using a single-shot spin-echo echo-planar sequence with tridirectional gradients (*b* values: 0, 500 s/mm^2^). Before the DWI scanning, array spatial sensitivity encoding technique was applied for array spatial sensitivity encoding technique (ASSET) scanning. The MR imaging parameters are listed in Table 1. Original images were transmitted to GE Medical Systems Functool 7.4.01d workstation for data processing. Two mathematical models of DWI, ADC and EADC, were performed for the analysis of diffusion-weighted images. Sb/S0 = EADC = exp [−(b × ADC)], *b* stands for the *b* value of the DWI sequence (i.e., *b* = 500 s/mm^2^ in our study), and Sb and S0 are the signal intensities on the diffusion-weighted image and the reference image, respectively.

**Table 1:** Parameters for DW imaging and MR imaging sequences

	Axial T2WI	Coronal-T2WI	IN-phase/OUT -phase imaging	DWI
Repetition time (ms)	∞	Minimum	4.1	2,000
Echo time (ms)	85.1–102	68.9–88.9	1.2/2.4	Minimum
Flip angle (°)	90	90	12	90
Bandwidth (kHz)	83.33	62.5	166.67	250
Matrix	320 × 224	288 × 288	256 × 180	128 × 128
Field of View (mm)	360–400	380–400	360–400	360–400
Slice thickness (mm)	5.0	5.0	5.0	5.0
Intersection gap (mm)	1.0	1.0	−2.5	1.0

The *b* values used for diffusion-weighted imaging were 0, 500 s/mm^2^

### Imaging Analysis

Two radiologists measured the EADC and ADC values of normal kidney and renal tumors independently. One has 14 years work experience in MRI, the other has 5 years work experience in functional MRI imaging (fMRI) of the kidney. Blind measurement was carried out on the data of lesions group. The original images were adjusted to their proper window width and position, and a region of interest (ROI) was defined based on in/out-phase and T2WI images. ROI setting in normal control group: on the cross-section of the bitmap with *b* = 0 s/mm^2^, ROI was delineated by hand, including all aspects of the renal parenchyma of each kidney, and kidney collecting system, renal sinus fat tissue were avoided. Then ADC map and EADC map of the same level were copied, measured and recorded.

**Fig. 1:**
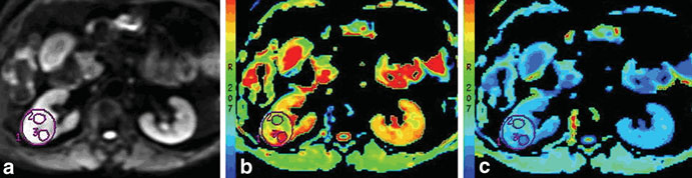
DWI map, EADC map and ADC map showing the parenchymal part and cystic part of tumor at multiple levels. **a** DWI map: the overall ROI1 of tumor appears as mixed signals, with diffusion-restricted high signals in parenchymal part (ROI3) and equal signals in cystic part (ROI2); **b** EADC map: ROI1 0.134, ROI2 0.0752, ROI3 0.256; **c** ADC map ROI1 2.102 × 10^−3^ mm^2^/s, ROI2 2.601 × 10^−3^ mm^2^/s, ROI3 1.372 × 10^−3^ mm^2^/s

In the group of patients, ROI was set as following: first, at all the transversal sections (*b* = 0 s/mm^2^) showing the appearance of the tumor, circular or oval ROI was adopted if the shape of tumor was regular, and manual delineated ROI was adopted to include the entire tumor if the shape of tumor was irregular; second, EADC and ADC values of each level were measured and the average value was regarded as EADC and ADC values for the whole tumor. Then the parenchymal part and cystic part of tumor were outlined at multiple levels and the mean values of EADC and ADC were obtained (Fig. 1). For tumors rich in fatty tissue, in addition to the above methods, the fatty part and non-fatty part of tumor were outlined respectively to obtain the their each mean value. For cystic lesions, ROI included the cystic wall as much as possible. There are no units of measurement for EADC value. Data were mean values averaged from two measurements.

### Statistical Analysis

All quantitative data are expressed as mean ± SD. Single-factor analysis of variance was performed to compare the differences in overall EADC and ADC values among normal group, renal cell carcinoma, angiomyolipoma and cysts. ROC curve was used to calculate diagnostic cutoff values, the sensitivity and specificity of EADC and ADC values in kidney cancer and angiomyolipoma, and area under the curve was underwent *U*-test analysis.

A *t* test was applied to the EADC and ADC between parenchymal tissue of renal cell carcinoma and non-fatty tissue of angiomyolipoma, between cystic renal cell carcinoma and simple renal cysts, and between clear cell carcinoma and non-clear cell carcinoma. The threshold of statistical significance for differences was set as *p* < 0.05.

SPSS for Windows version 16.0.0 (SPSS, Inc, Chicago, IL, USA) statistical software was used for statistical analysis.

## Results

### Lesion Characteristics

There were no obvious artifacts found in the 90 patients and 20 healthy volunteers in conventional MRI and DWI images. Enhanced MRI scanning was performed in 22 patients. Their original images were used for reconstruction and EADC and ADC map measurement. A total of 101 lesions in 90 patients were measured. A total of 79 lesions were confirmed by pathology 14.23 days after the MRI examination, 43 patients underwent radical resection, 29 patients underwent nephron-sparing surgery, and 7 patients were confirmed by biopsy. There were 45 lesions in 44 patients of renal cell carcinoma. One patient had a tumor in each kidney, with lesion diameter of 5.2 cm in right kidney and 2.2 cm in left kidney. Radical surgery was performed on right kidney for this patient, and the nephron-sparing surgery was performed 3 months later. Among all patients, there were 37 cases of renal cell carcinoma, 3 cases of chromophobe cell tumor, 2 cases of papillary renal cell carcinoma, and 1 case of renal metastatic adenocarcinoma, 2 cases of cystic renal cell carcinoma, 22 cases of renal angiomyolipoma, and 12 cases of simple renal cysts. In one patient with multiple renal angiomyolipomas, only two large lesions were removed, and the remaining five lesions were in the process of follow-up (followed up every year). A total of 11 renal angiomyolipoma lesions and 11 renal cysts lesions with typical performances have been followed up for 6–8 months (Table 2).

**Table 2:** Lesion characteristics

Pathology	Number of patient	Number of lesions	Mean size (cm)	M	F	Median age	Confirmed by pathology
Renal cell carcinoma	44	45	4.41 ± 1.66	31	13	52	45
Clear cell carcinoma	36	37	4.36 ± 1.67	25	11	51	37
Chromophobe cell carcinoma	3	3	4.76 ± 1.66	1	2	48	3
Papillary renal cell carcinoma	2	2	5.75 ± 2.05	2	0	41	2
Metastatic adenocarcinoma	1	1	4.3	1	0	51	1
Cystic renal cell carcinoma	2	2	2.85 ± 0.49	2	0	53	2
Angiomyolipoma	26	33	4.85 ± 3.03	15	21	43	22
Cyst	20	23	3.98 ± 1.77	11	12	40	12
Normal kidney	20	20	–	10	10	46	–

### Image Performance

**Fig. 2:**
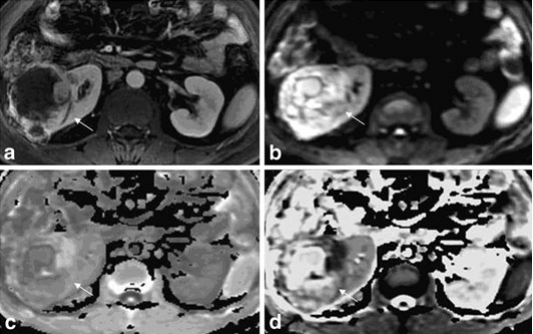
Right renal clear cell carcinoma, grade II with in a patient of 63 years old (male). **a** Enhanced scan in medulla phase, the substantial part of the right kidney lesions showed irregular peripheral enhancement, and the central part with necrosis and cystic degeneration showed no enhancement. **b** DWI lesions showed mixed high signal intensity in both the tumor parenchyma and central cystic area. **c** The EADC lesions showed peripheral high signal and central low signal, which was in accordance with what was displayed by enhanced scanning. In the background of gray kidneys, the lesion was highlighted with a clear internal structure. The high signal indicates the substantial part of the tumor. **d** ADC lesions showed mixed high signal, with high signal in central necrosis part and low signal in surrounding parenchyma part. Compared with the enhanced scan and EADC map, the peripheral part of the kidney was less legible by ADC map. The abdominal intestinal signal was cluttered. Compared with the ADC map, EADC map showed much clean background and much clear image

**Fig. 3:**
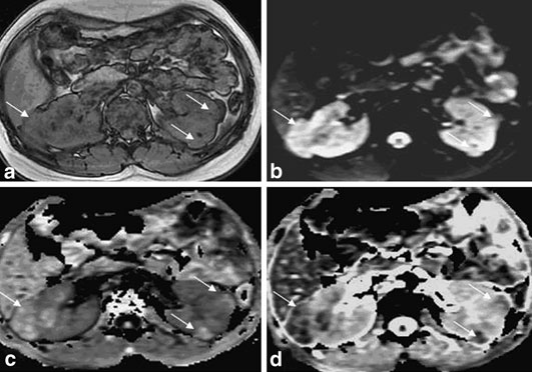
Multiple renal angiomyolipomas in a 16 years old girl. **a** T1WI in-phase showed irregular prominence of signal image within *right* kidney, and multiple prominent extrarenal semicircular signal intensity within *left* kidney, with ill-defined border. **b** DWI lesions showed uneven slightly higher signal intensity. **c** The EADC map showed uneven high signal with a clear boundary, and the lesions were highlighted in the context of gray kidney. **d** ADC map showed uneven low signal intensity with cluttered kidney background

**Fig. 4:**
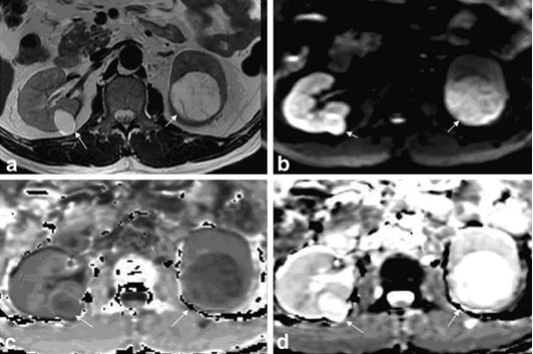
Kidney cyst in a 52 years old woman. **a** T2WI showed two cysts (*arrow*) in left and right kidney, respectively. Visible thin line separator can be seen in the *left* kidney cyst. **b** DWI showed high signal intensity. **c** EADC showed low signal intensity. The separator within the *left* kidney lesions showed slightly higher signal intensity. **d** ADC showed significantly high signal intensity. Part of the structure within the *left* kidney lesions was covered

The EADC map had a distinct advantage over ADC map. Among 45 lesions of renal cell carcinoma from 44 patients, 12 lesions showed significantly higher DWI performance, higher EADC signal intensity and lower ADC signal intensity. In 33 lesions with mixed high signal in DWI performance, the EADC showed mixed high signal in the background of gray and white kidney, which highlighted the lesions and exhibited a clear structure within lesions. The higher the signal of the area, the higher the EADC value was. The high signal parenchyma within tumor displayed by EADC was in accordance with displayed by enhanced scanning. ADC map showed mixed low signal, and compared with EADC map, ADC map showed relative cluttered background and less clear internal structure within tumor lesions (Fig. 2). Among 33 lesions of renal hamartoma tumor among 23 patients, 26 lesions showed significantly uneven low signal in DWI performance, uneven high signal in EADC performance and low signal shadow in ADC performance. Other seven lesions showed uneven high signal in DWI performance, high signal in EADC performance and low signal in ADC performance (Fig. 3). Renal cysts showed uniform high signal intensity in DWI performance, uniform low signal in EADC performance and high signal in ADC performance. In two cases of Bosniak cyst type II of this study, DWI showed slightly higher signal intensity, and EADC showed low signal with the visible separation. The significantly higher signal displayed by DWI had concealed part of lesion within the structure (Fig. 4).

### Comparisons Between Normal Kidney and Renal Cell Carcinoma, Angiomyolipoma, Cyst in ADC and EADC Values

**Fig. 5:**
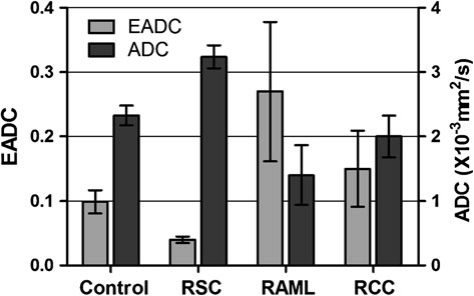
Different measured value of EADC and ADC tested in the RCC, RSC, RAML and normal renal parenchyma. There were statistical differences of EADC and ADC values in renal cell carcinoma, cysts, angiomyolipoma and the normal renal parenchyma (*p* < 0.05)

When *b* = 500 s/mm^2^ (3.0 T GE MRI), there were statistical differences in EADC and ADC values between overall renal cell carcinoma, cysts, angiomyolipoma and the normal renal parenchyma (*p* < 0.05). The value of EADC was highest in renal angiomyolipoma (0.272 ± 0.061, range 0.092–0.486), followed by renal cell carcinoma (0.150 ± 0.059, range 0.12–0.632) and normal renal parenchyma (0.099 ± 0.016, range 0.073–0.138), while the value of EADC was the lowest in renal cysts (0.040 ± 0.005, range 0.029–0.051). Correspondingly, the value of ADC was highest in renal cysts [(3.237 ± 0.179) × 10^−3^ mm^2^/s, range (2.980–3.770) × 10^−3^ mm^2^/s], followed by the normal renal parenchyma [(2.326 ± 0.153) × 10^−3^ mm^2^/s, range (2.010–2.620) × 10^−3^ mm^2^/s] and renal cell carcinoma [(2.001 ± 0.322) × 10^−3^ mm^2^/s, range (1.120–2.640) × 10^−3^mm^2^/s], while the value of ADC was lowest in angiomyolipoma [(1.402 ± 0.461) × 10^−3^ mm^2^/s, range (0.767–2.390) × 10^−3^ mm^2^/s] (Fig. 5). The difference is mainly caused by variations in organ structure and diffusion of water molecules.

### Comparisons Between Renal Cell Carcinoma and Angiomyolipoma

**Fig. 6:**
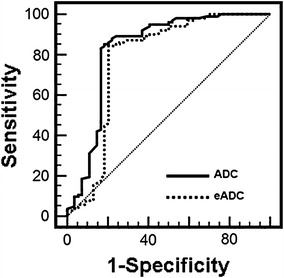
Receiver operating characteristic (ROC) curve of the threshold EADC (0.192) and ADC value (1.66 × 10^−3^ mm^2^/s) was used for differentiating RCC from RAML. The sensitivity and specificity are 84.6 % in EADC and 81.1 % in ADC, respectively. No significant difference was found in area under the ROC curve between EADC and ADC values by *U*-test analysis (*p* > 0.05)

**Fig. 7:**
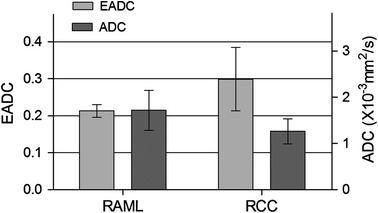
Different measured value of EADC and ADC tested in the parenchyma part of RCC and the non-fatty part of RAML. There was a significant difference in EADC and ADC between the parenchyma part of RCC and the non-fatty part of RAML, respectively, (*p* < 0.05)

There was statistically significant difference between overall EADC value in renal cell carcinoma (0.150 ± 0.059) and EADC in angiomyolipoma (0.270 ± 0.108) (*p* < 0.05). Correspondingly, there was also statistically significant difference between ADC in renal cell carcinoma [(2.001 ± 0.322) × 10^−3^mm^2^/s] and in angiomyolipoma [(1.402 ± 0.461) × 10^−3^mm^2^/s] (*p* < 0.05). When EADC value was more than 0.192, and ADC value was less than 1.66 × 10^−3^mm^2^/s, the sensitivity and specificity of diagnosis of renal cell carcinoma was 84.6 and 81.1 %, respectively (Fig. 6). No significant difference was found in area under the ROC curve between EADC and ADC values by *U*-test analysis (*p* > 0.05), which might be due to the correlation between EADC and ADC (EADC = exp[−(b × ADC)]). Renal cell carcinoma with typical performance is not easy to be misdiagnosed as angiomyolipoma. The greatest difference between atypical angiomyolipoma and renal cell carcinoma lies in their different renal parenchymal structures. There was statistical difference in EADC between parenchyma of renal cell carcinoma (0.299 ± 0.086) and the non-fatty part of angiomyolipoma (0.179 ± 0.088) (*p* < 0.05). Correspondingly, there was statistical difference in ADC between parenchyma of renal cell carcinoma [(1.264 ± 0.271) × 10^−3^ mm^2^/s] and the non-fatty part of angiomyolipoma [(1.717 ± 0.431) × 10^−3^ mm^2^/s] (*p* < 0.05) (Fig. 7). Our measurement method is easier to operate and was more suitable for the differential diagnosis in atypical lesions.

### Comparisons Between Renal Cysts and Cystic Tumors

**Fig. 8:**
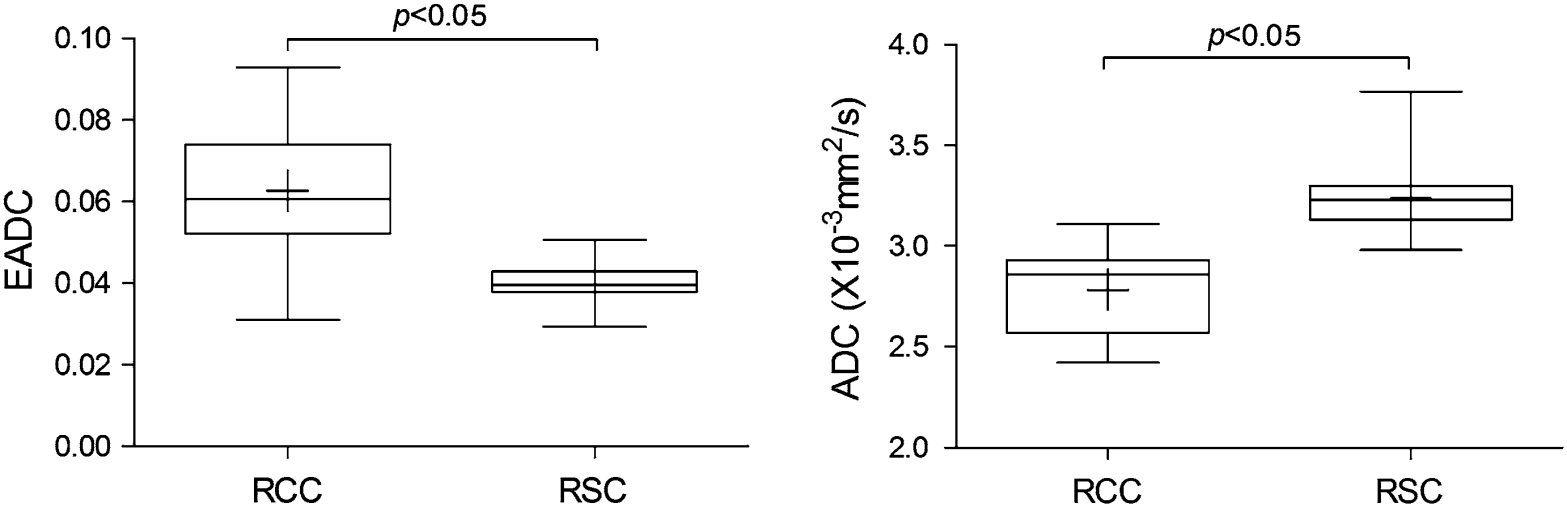
Groupwise comparison of EADC (**a**) and ADC (**b**) value of cystic lesion in two groups based on pathological categories. A statistical difference between RCC and RSC was observed in subjects with EADC (*p* < 0.05) and ADC (*p* < 0.05), respectively, (*RCC* renal cell carcinoma, *RSC* renal simple cyst, + median)

There were 28 obvious cystic necrosis in 45 tumor lesions, and the EADC value in simple cysts (0.040 ± 0.005) was significantly lower than that in tumors with cystic necrosis (0.063 ± 0.014) (*p* < 0.05). Similarly, there was statistically significant difference in ADC value between the cysts (3.237 ± 0.179 × 10^−3^ mm^2^/s) and tumors with cystic necrosis [(2.784 ± 0.204) × 10^−3^ mm^2^/s] (*p* < 0.05) (Fig. 8).

### Comparisons Between Clear Cell Carcinoma and Non-Clear Cell Carcinoma

**Fig. 9:**
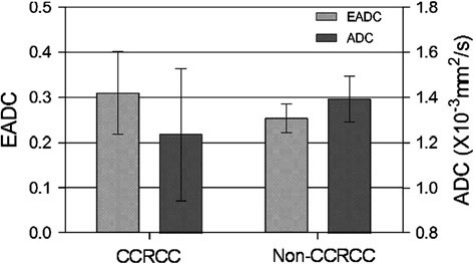
Different measured value of EADC and ADC value tested in the parenchyma of CCRCC and Non-CCRCC. There was a significant difference in EADC and ADC between the CCRCC and non-CCRCC (*p* < 0.05)

Exponential apparent diffusion coefficient value in clear cell carcinoma and non-clear cell carcinoma was 0.309 ± 0.092 and 0.253 ± 0.032, respectively. ADC value in clear cell carcinoma and non-clear cell carcinoma was (1.234 ± 0.292) × 10^−3 ^mm^2^/s and (1.391 ± 0.102) × 10^−3^ mm^2^/s, respectively. There were significant differences in EADC and ADC values between clear cell carcinoma and non-clear cell carcinoma (*p* < 0.05) (Fig. 9).

## Discussion

This study firstly explored the application of EADC value in the high magnetic field strengths (3.0 T MRI) in the kidney lesions. The signal-to-noise ratio of DWI was significantly improved by 3.0 T MRI compared with 1.5 T MRI under the same *b* value [21]. In this study, ASSET technology reduced the scan time and motion artifacts under the condition that resolution was guaranteed [22].

In previous reports, the researchers mainly studied the application of ADC value in space-occupying kidney disease. ADC value has been used in differentiating among the various subgroups of renal masses, predicting nuclear grade and histological subtype in renal cell carcinoma and differentiating angiomyolipoma from CCRCC with minimal fat [23–25]. However, studies on differentiating diagnosis among the space-occupying kidney disease using EADC value are limited. EADC is an indicator of innovative methods of MRI-diffusion data analysis that merges the advantages of DWI and ADC maps. Compared with ADC, EADC reflects altered water diffusion with out T2 shine-through effects.

The EADC and ADC values are influenced by chosen ROI to a large extent. Multiple ROI was performed to measure multiple levels within lesions, and the mean or minimum values were calculated in most of the reports [23, 26–28]. But only the parenchymal part of central level in tumor was measured instead of necrotic, hemorrhagic or cystic part in some reports [29, 30]. Wang et al. measured the ROI on every level of the parenchymal part of the tumor, and the minimal ROI was adopted [31]. However, we measured the entire tumor layer by layer, including parenchymal part and necrotic, cystic and hemorrhagic part, and their mean value was equivalent to the volume. The value of EADC and ADC in this study reflects the degree of diffusion of water molecules in overall tumor rather than the local neoplasm. At the same time, the parenchymal part of the tumor was also measured.

The difference of *b* value also affects the size of the ADC value. Since there is no unified standard of *b* value used in kidney disease studies, the adopted *b* value ranges from 0 to 1000 mm^2^/s. In theory, the greater the *b* value, the better it reflects the diffusion of water molecules within tissue. However, greater *b* value increases the scan time, decreases the signal-to-noise ratio, and increases the risk of motion artifacts. Given a variety of factors, we and many other researchers [16, 27, 28] selected a *b* value of 500 s/mm^2^, which produced images with high signal-to-noise ratio and fewer artifacts (the artifacts only showed in one case of the patient group). The breath-hold time of whole kidney scanning (24-layer) was 14 s with the *b* value of 500 s/mm^2^, which was not difficult for the patients. Our results of ADC and EADC values were stable and comparable.

Our results show when *b* is 500 mm^2^/s with high magnetic field strength (3.0 T), the EADC value of normal kidney is 0.099 ± 0.016 (range 0.073–0.138). Correspondingly, ADC value of normal kidney is (2.326 ± 0.1531) × 10^−3^ mm^2^/s (range (2.010–2.620) × 10^−3^ mm^2^/s). Manenti et al. [18] reported when b was 500 mm^2^/s in 3.0 T MRI, the ADC value of normal kidney was (2.35 ± 0.31) × 10^−3^ mm^2^/s (range 2.10–2.61 × 10^−3^ mm^2^/s), and this results are quite consistent with ours. Wang et al. [31] reported in 3.0 T MRI when *b* value was 500 mm^2^/s that the ADC value of normal kidney was (2.455 ± 0.238) × 10^−3^ mm^2^/s. Most other studies were conducted by the 1.5 T MRI and the measured ADC values ranged from (2.08 ± 0.22) × 10^−3^ mm^2^/s to (2.67 ± 0.29) × 10^−3^ mm^2^/s, using the *b* value from 300 to 1000 mm^2^/s. The variations in ADC values are caused by differences in *b* values and scanning parameters [32–35].

EADC value could help the differential diagnosis between angiomyolipoma and renal cell carcinoma. Renal angiomyolipoma is the most common benign tumor. The most difference in treatment between angiomyolipoma and renal cell carcinoma is that small asymptomatic angiomyolipoma only needs to be followed up regularly instead of surgery, but small renal cell carcinoma requires resection. Angiomyolipoma with less adipose tissue can be easily misdiagnosed as renal cell carcinoma, while renal cell carcinoma containing adipose tissue can be easily misdiagnosed as angiomyolipoma. Our results show that in the whole tumor, the EADC value of renal angiomyolipoma (0.270 ± 0.108) is higher than that of renal cell carcinoma (0.150 ± 0.059). Correspondingly, ADC value of angiomyolipoma [(1.402 ± 0.461) × 10^−3^ mm^2^/s] is lower than that of renal cell carcinoma [(2.001 ± 0.322) × 10^−3^ mm^2^/s]. Significant differences were found in EADC and ADC values between angiomyolipoma and renal cell carcinoma, which is consistent with the findings reported in the literature [28, 29]. The low ADC value of angiomyolipoma is affected by ADC value in fatty part [(0.651 ± 0.154) × 10^−3^ mm^2^/s]. When EADC value is greater than 0.192, and ADC value is less than 1.66 × 10^−3^ mm^2^/s, the sensitivity and specificity of diagnosis of renal cell carcinoma were 84.6 and 81.1 %, respectively. In this group of patients, one case with chromophobe containing adipose tissue was misdiagnosed as angiomyolipoma. The EADC value in non-fatty part of tumor was 0.218, and ADC value was 1.53 × 10^−3^ mm^2^/s, indicating high possibility of renal cell carcinoma. Razek et al. [35] set the threshold of ADC value as 1.84 × 10^−3^ mm^2^/s in 1.5 T MRI when b value was 800 mm^2^/s, and the sensitivity and specificity for diagnosis of benign and malignant lesions was 89 and 89 %, respectively. Sandrasegaran [23] set the threshold of ADC value as 2.19 × 10^−3^ mm^2^/s in 1.5 T MRI when *b* value was 800 mm^2^/s, and the sensitivity and specificity for diagnosis of benign and malignant lesions was 100 and 88 %, respectively. The thresholds in these authors’ studies is higher than that in our study, and this difference is mainly caused by significantly lower ADC values measured in 3.0 T MRI than in 1.5 T MRI [21].

At the same time, when we measured tumors with adipose tissue, the fatty part and non-fatty part were measured respectively. The EADC and ADC values of the non-fatty part of angiomyolipoma were 0.213 ± 0.217 and (1.717 ± 0.431) × 10^−3^ mm^2^/s, respectively. The EADC and ADC values of the parenchymal part of renal cell carcinoma were 0.299 ± 0.086 and (1.264 ± 0.271) × 10^−3^ mm^2^/s, respectively. There is significant difference in EADC and ADC values between angiomyolipoma and renal cell carcinoma. The EADC values in angiomyolipoma were lower than that in renal cell carcinoma, suggesting that the intensity of tumor cells in parenchymal part of angiomyolipoma is less than that of malignant tumor cells, which is very important in the differential diagnosis between renal cell carcinoma and atypical angiomyolipoma containing less adipose tissue.

We found that simple cysts have significantly lower EADC value and higher ADC value than in tumors with cystic necrosis. The simple renal cysts are mainly composed of fluid, leading to relatively free diffusion of water molecules. In the cyst fluid within tumors with cystic necrosis, there are still a few cellular components, with greater viscosity than the cysts, so the EADC value is higher than in cysts. Our results are consistent with the current literature reported [29, 34, 36].

The EADC values in clear cell carcinoma are higher than those in non-clear cell carcinoma, while ADC values in clear cell carcinoma are lower than those in non-clear cell carcinoma, and these differences are statistically significant, indicating higher restricted diffusion in clear cell carcinoma than in non-clear cell carcinoma. Paudyal et al. [16] found a significant difference in ADC values between clear cell carcinoma (*n* = 25, ADC = (1.59 ± 0.55) × 10^−3^ mm^2^/s) and non-clear cell carcinoma [(papillary carcinoma (*n* = 6) and chromophobe carcinoma (*n* = 1), ADC = (6.72 ± 1.85) × 10^−3^ mm^2^/s], which is consistent with our findings. Razek et al. [36] thought that the ADC values in clear cell carcinoma (*n* = 19, ADC = (1.74 ± 0.12) × 10^−3 ^mm^2^/s) are higher than those in non-clear cell carcinoma (papillary carcinoma (*n* = 6), ADC = (1.65 ± 0.26) × 10^−3^ mm^2^/s, chromophobe carcinoma (*n* = 8), ADC = (1.44 ± 0.0.12) × 10^−3^ mm^2^/s). Wang et al. [31] thought ADC value in clear cell carcinoma (*n* = 49, ADC = 1.849 × 10^−3^ mm^2^/s) was significantly higher than that in papillary carcinoma (*n* = 22, ADC = 1.087 × 10^−3^ mm^2^/s) and chromophobic cell carcinoma (*n* = 14, ADC = 1.307 × 10^−3^ mm^2^/s). Sandrasegaran et al. [23] found no significant difference in ADC value between clear cell carcinoma (*n* = 17, ADC = (1.85 ± 0.23) × 10^−3^ mm^2^/s) and non-clear cell carcinoma (papillary RCC (*n* = 5), chromophobe RCC (*n* = 1), ADC = (1.97 ± 0.14) × 10^−3^ mm^2^/s). However, in these studies as well as in our study, the number of non-clear cell carcinoma is small, and more cases are needed to accumulate for further evaluation of its value and reliability.

According to the formula: EADC = exp [−(b × ADC), EADC shows no advantage and difference compared with ADC, but showed obvious advantage over ADC on the image. Compared with ADC map, EADC map shows better background suppression, for example, the majority of renal cell carcinoma lesions showed mixed high signal in the EADC performance with clear lesion border. The higher the signal of region, the higher the EADC value and the lower the ADC value are, indicating tumor tissues with the most substantial part and most densely populated tumor cells [18].

Although we explored an interesting means to display DWI data based on EADC values, its utility in clinical practice remains to be determined. A small preliminary study showed that use of EADC value could reliably differentiate acute infarcts (less than 5 days old) from infarcts more than 10 days old [37]. The author believed that EADC value may provide help when the distinction between acute and nonacute infarction cannot be determined on clinical grounds. Future studies will address the clinical utility of EADC in space-occupying kidney disease and its accuracy and precision in size and shape of kidney masses.

The limitations of this study are as follows: the sample size of kidney cancer was not large enough, and only a few cases of kidney cancer of rare types were collected. Classification was not performed on low-level and advanced clear cell carcinoma. The cases of complicated renal cysts in this study were also limited. Whether EADC can be used in typing of the Bosniak cyst remains further investigation.

In conclusion, EADC map shows more intuitively in the internal structure of the kidney tumor than the ADC map dose, and is also in accordance with the observation habits of the clinicians. EADC can be used as an effective imaging method for tumor diagnosis.
